# Hodgkin Lymphoma-Derived Extracellular Vesicles Change the Secretome of Fibroblasts Toward a CAF Phenotype

**DOI:** 10.3389/fimmu.2018.01358

**Published:** 2018-06-18

**Authors:** Bastian Dörsam, Teresa Bösl, Katrin S. Reiners, Sabine Barnert, Rolf Schubert, Olga Shatnyeva, Paola Zigrino, Andreas Engert, Hinrich P. Hansen, Elke Pogge von Strandmann

**Affiliations:** ^1^Clinic for Hematology, Oncology and Immunology, Experimental Tumor Research, Center for Tumor Biology and Immunology, Philipps University, Marburg, Germany; ^2^Department of Internal Medicine, University Hospital of Cologne, Cologne, Germany; ^3^Department of Pharmaceutical Technology and Biopharmacy, Albert-Ludwigs-University, Freiburg, Germany; ^4^Department of Dermatology, University Hospital of Cologne, Cologne, Germany

**Keywords:** Hodgkin lymphoma, extracellular vesicles, tumor microenvironment, cancer-associated fibroblasts, NF-κB-signaling

## Abstract

Secretion of extracellular vesicles (EVs) is a ubiquitous mechanism of intercellular communication based on the exchange of effector molecules, such as growth factors, cytokines, and nucleic acids. Recent studies identified tumor-derived EVs as central players in tumor progression and the establishment of the tumor microenvironment (TME). However, studies on EVs from classical Hodgkin lymphoma (cHL) are limited. The growth of malignant Hodgkin and Reed–Sternberg (HRS) cells depends on the TME, which is actively shaped by a complex interaction of HRS cells and stromal cells, such as fibroblasts and immune cells. HRS cells secrete cytokines and angiogenic factors thus recruiting and inducing the proliferation of surrounding cells to finally deploy an immunosuppressive TME. In this study, we aimed to investigate the role of tumor cell-derived EVs within this complex scenario. We observed that EVs collected from Hodgkin lymphoma (HL) cells were internalized by fibroblasts and triggered their migration capacity. EV-treated fibroblasts were characterized by an inflammatory phenotype and an upregulation of alpha-smooth muscle actin (α-SMA), a marker of cancer-associated fibroblasts. Analysis of the secretome of EV-treated fibroblast revealed an enhanced release of pro-inflammatory cytokines (e.g., IL-1α, IL-6, and TNF-α), growth factors (G-CSF and GM-CSF), and pro-angiogenic factors such as VEGF. These soluble factors are known to promote HL progression. In line, ingenuity pathway analysis identified inflammatory pathways, including TNF-α/NF-κB-signaling, as key factors directing the EV-dependent phenotype changes in fibroblasts. Confirming the *in vitro* data, we demonstrated that EVs promote α-SMA expression in fibroblasts and the expression of proangiogenic factors using a xenograft HL model. Collectively, we demonstrate that HL EVs alter the phenotype of fibroblasts to support tumor growth, and thus shed light on the role of EVs for the establishment of the tumor-promoting TME in HL.

## Introduction

Hodgkin lymphoma (HL) is a rare cancer usually arising in the lymph nodes, which was initially described by Thomas Hodgkin (1). Mainly, two distinct entities are described for HL, the classical Hodgkin lymphoma (cHL) accounting for 95% of all cases, and a rare nodular lymphocyte predominant HL form (2).

A unique characteristic of classical HL is that the malignant Hodgkin and Reed–Sternberg cells (HRS cells) account for only 1% of the tumor tissue, which is composed of a massive infiltrate of reactive cells (lymphocytes, fibroblasts, and cells of the innate immune system) (3). Typically, HRS cells are surrounded by impaired T cells, forming a T cell rosette, which impedes a direct interaction with other cells. Thus, crosstalk *via* soluble factors and a complex network of chemokine/cytokine interactions facilitates the establishment of a tumor-supportive environment (4). HRS cells usually arise from mature B cells but undergo a severe alteration during progression to malignant cells concomitant with loss of characteristic markers for B cells/cells of the hematopoietic system (5). The events involved in genesis of malignant HRS cells are partially understood; however, the most frequent changes result in consecutive activation of the NF-κB-signaling pathway and deregulation of other pathways, including JAK/STAT, MAPK/ERK, NOTCH1, and PI3K/AKT. Although HRS cells are considered the master regulator of the inflammatory response in the lymphoid tissue of HL, survival of the few malignant cells is likely dependent on the tumor microenvironment (TME) and interaction with non-malignant cells (3). The HL-specific TME is constituted of many different cell types, including immune cells, such as lymphocytes, plasma cells, neutrophils, eosinophils, and mast cells as well as fibroblasts. Expression of a variety of cytokines and chemokines facilitate the attraction of immune cells and the establishment of this tumor-promoting milieu and, therefore, has been extensively studied in the past years (6, 7). HL cells exploit different mechanisms to escape from immune surveillance, including the inhibition of effector cells, e.g., *via* secretion of immune suppressive molecules, such as TARC, MICA, and BAG6, ligands for receptors (CCR4, NKG2D, NKp30, respectively) expressed on a subset of T cells and NK cells (8–11).

Recently, the relevance of extracellular vesicles (EVs) for the intercellular crosstalk and the establishment of a tumor-promoting microenvironment was raised in several studies (12, 13).

Extracellular vesicles are a central part of intercellular communication allowing cells to interact with close and distant cells *via* the delivery of signal molecules. In detail, EVs play a crucial role in the diverse interactions in the tumor-supportive ME. The smallest EV-subpopulation with a diameter of 50–150 nm is commonly referred to as exosomes, which are generated *via* the endocytic pathway and carry parent cell-specific molecules. These molecules include proteins, DNA, noncoding RNAs, and miRNAs/mRNAs (13). The chaperone HSP70 and tetraspanins CD9, CD63, and CD81 are present on EV-subpopulations and commonly used as markers (14).

Here, we investigate the EV-mediated interplay of HL cells and fibroblast. In detail, we report that HL cells and fibroblasts interact in a bi-directional manner, changing migratory properties and, most interestingly, encouraging the transition of healthy fibroblasts to a CAF phenotype concomitant with alteration of their inflammatory secretome.

## Materials and Methods

### Cell Culture

The human HL cell line KM-H2 (Deutsche Sammlung von Mikroorganismen und Zellkulturen) as well as the human primary fibroblast cell line HDF_n_ (American Type Culture Collection) were maintained in DMEM GlutaMAX or RPMI 1640 (Thermo Fisher Scientific) supplemented with 10% fetal calf serum (FCS) and antibiotics (100 U/ml penicillin and 100 µg/ml streptomycin) at 37°C with 5% CO_2_.

### Isolation of EVs and Quantification of EV Protein Cargo

Cells were cultivated in EV-depleted medium for 48 h followed by EV-isolation *via* sequential ultracentrifugation as previously described (15). In brief, supernatants were centrifuged for 10 min at 300 × *g*, 10 min at 3,000 × *g*, and 30 min at 10,000 × *g*. Subsequently, the supernatant of the 10,000 × *g* fraction was centrifuged for 90 min at 100,000 × *g* and the obtained pellet washed with PBS at the same speed for 90 min. After resuspension of the pellet in PBS, protein content was determined using the BCA protein assay kit (Thermo Scientific) and a SpectraMax M4 (Molecular Devices). Samples were stored at −80°C for further analysis.

### EV-Depletion of Medium and Cultivation of Cells With Isolated EVs

Extracellular vesicles were removed from the medium by ultracentifugation (90 min at 100,000 *g*). The pellet containing EVs was carefully discarded. Cells were cultivated in EV-depleted medium with purified EVs as indicated.

### Nanoparticle Tracking Analysis (NTA)

Number and size distribution of isolated EVs were estimated by the means of NTA. EVs were diluted 1:1,000 with PBS (Biochrom). Five repeated measurements of 60 s with an infusion rate of 40 were recorded consecutively and analyzed using a Nanosight NS300 with the NTA 3.0 software (Malvern Instruments).

### Flow Cytometry

Adherent cells were harvested using Accutase (Life Technologies). For flow cytometric assessment, cells were stained with different concentrations of DiO (AAT Bioquest) for 5 min at 37°C. Isolated vesicles were processed for flow cytometry as described: 100 µg (≈1 × 10^9^) EVs were coupled to 1 × 10^5^ 4.5 µm polystyrene beads (Polysciences) in PBS over night at 4°C for assessment of surface proteins (15). After blocking with 2% bovine serum albumin (BSA) for 1 h at 25°C under shaking, molecules of interest were either probed with labeled Annexin V, respectively a labeled primary PE-labeled antibody against CD30 (BioLegend, 333906, 1:100) or primary antibodies against CD9 (BioLegend, 312102, 1:100), CD63 (BioLegend, 353013, 1:100), or CD81 (BioLegend, 349501, 1:100), and a secondary goat anti-mouse-PE antibody (BioLegend, 405705, 1:100). Antibody incubation was performed for 30 min on ice under exclusion of light in FACS buffer (PBS with 0.2% BSA, 0.2% sodium azide). Samples were analyzed with a FACS Calibur (Becton Dickinson).

### Electron Microscopy

Extracellular vesicles were isolated from the supernatant of KM-H2 cells and resuspened in PBS. 3 µl of the EV solution was transferred onto a copper grid (Quantifoil S7/2 Cu 400 mesh, carbon films; Quantifoil Micro Tools). After removal of excess liquid, the copper grid was snap-frozen by immersion into liquid ethane. Samples were analyzed with a transmission electron microscope (Leo 912 Ω-mega) at −174°C. The device was operated at 120 kV and images recorded with a 6,300- to 12,500-fold magnification.

### SDS-PAGE and Immunoblot Analysis

Adherent cells were harvested with Accutase and whole cell lysates of the cell pellet prepared using a buffer containing 50 mM Tris pH 8.0, 150 mM NaCl, 0.5% Triton X-100, 0.5% sodium deoxycholate, protease, and phosphatase inhibitors (Roche). 100 µl of the buffer was used to lyse 1 × 10^6^ cells for 5 min under rotation at room temperature (RT). Subsequently, the samples were centrifuged at 14,000 *g* for 10 min at 4°C, the pellet removed and the lysate stored at −20°C if it was not processed directly upon preparation. Protein content was determined using the BCA protein assay kit (Thermo Fisher Scientific). 20 µg of protein per sample were heated in Laemmli sample buffer to 96°C for 10 min and subjected on a 10% SDS-PAGE. After separation, proteins were transferred to a 0.2 µm nitrocellulose membrane (GE Healthcare) with a wet blot chamber (BioRad). After blocking with 5% non-fat dry milk in TBS-T (137 mM NaCl, 50 mM Tris–Cl, 0.05% Tween-20, pH 7.4), the membrane was probed with the desired primary antibodies against β-Actin (Abcam, ab6276, 1:15,000), CD9 (Santa Cruz Biotechnology, sc-13118, 1:100), CD63 (Invitrogen, 10628D, 1:500), or CD81 (BioLegend, 349501, 1:500) for 2 h at RT and then washed three times with TBS-T. Incubation with the appropriate horseradish peroxidase-conjugated secondary antibody (Cell Signaling Technology, 7076, 1:2,500) was performed for 1 h at RT. Proteins were detected *via* enhanced chemiluminescence using Pierce ECL Western Blotting Substrate (Thermo Fisher Scientific).

### Immunofluorescence

The phenotype switch of fibroblasts after exposure to EVs (scratch assay) was analyzed by probing α-SMA as a marker for activated fibroblasts. Cells were grown on cover slips and fixed with 4% paraformaldehyde for 15 min at RT, washed thrice with PBS, and permeabilized with 1% Triton X-100 in PBS for 30 min at RT. Subsequently, washed cells were blocked with 10% FCS and 0.2% Tween-20 in PBS for 30 min at RT and incubated with a FITC-conjugated α-SMA antibody (Sigma-Aldrich, F3777, 1:250) for 1 h at RT under exclusion of light. Nuclei were stained with 1 µg/ml DAPI (Roche). Cover slips were mounted on microscope slides using VECTASHIELD Antifade Mounting Medium for Fluorescence (VECTAMicroscopic analysis was performed using an Olympus IX51 with the imaging software CellSens).

Extracellular vesicles were visualized by staining of parental HL cells with 1 µM DiO for 5 min at 37°C prior to seeding. DiO-positive (DiO^+^) vesicles were harvested *via* sequential ultracentrifugation. 100 µg/ml DiO^+^ EVs were added to fibroblast cell growing on cover slips for 2 days. Cells were processed in the way described above. The plasma membrane of fibroblasts was stained with CellMask Deep Red Plasma Membrane Stain [Invitrogen according to the manufacturer’s protocol and the nuclear dye Hoechst 33342 (Sigma-Aldrich, B2261, 1:5,000)]. Followed by washing with PBS, internalization of EVs by target cells was evaluated with the confocal microscope Leica TCS SP8.

### Cell Viability Assay (XTT Assay)

The impact of HL EVs isolated from KM-H2 cell culture on the proliferation of HDF_n_ cells and *vice versa* was probed using the XTT assay (AppliChem). 6 × 10^3^ KM-H2 or 1 × 10^5^ HDF_n_ cells per well were seeded on a 96-well plate. Cells were incubated with the amount of EVs and time period indicated in the according figure. The XTT staining solution was prepared according to the manufacturer’s protocol, 50 µl staining solution added to each well containing 100 µl growth medium, and incubated for 2 h at 37°C. Absorbance was then measured with an Infinite M1000 microplate reader (Tecan) at a wave length of 475 and 660 nm as reference.

### Migration Assays

Migration of HL cells was studied in a 24-well Boyden chamber with 8.0 µm pores (Falcon/Fisher Scientific). 1 × 10^6^ HL cells were transferred into the upper compartment, while crude fibroblast supernatant or medium (with serum, EV depleted) containing 100 µg/ml EVs were placed in the lower compartment. According to the NTA data, a protein concentration of 100 µg/ml corresponds to about 1 × 10^9^ Hodgkin cell-derived EVs/ml. Migrated cells were counted after an incubation time of 26 h.

The scratch assay was performed to monitor the migration of fibroblasts. 1.5 × 10^5^ HDF_n_ cells/well were seeded in a 24-well Falcon plate. The cell layer was impaired with a scratch and 100 µg/ml HL EVs or medium added. For every condition, cells were seeded in triplicates and two spots per well were monitored with images being recorded in an interval of 15 min for 24 h. For analysis, the scratch width was determined with ImageJ (National Institutes of Health) after 0, 3, 12, and 21 h, wound closure was calculated with the following formula:

$$\%\text{wound closure=1}-\left(\frac{\text{scratch width}t_{x\text{h}}}{\text{scratch width}t_{\text{0h}}}\right)\times 100\text{.}$$

Directed migration was studied with a chemotaxis assay performed in Neuroprobe ChemoTx plates. Migration of Calcein AM (MoBiTe)-labeled HDF_n_ cells toward 40 µg/ml, 150 mg/ml HL EVs or medium (triplicates), was assessed after 2 h *via* fluorescence detection with an Infinite M1000 microplate reader (Tecan).

### Cytokine Array

Chemokines/cytokines in the supernatant of fibroblast cells were quantified using the Human Cytokine Array/Chemokine Array 64-Plex from Eve Technologies and a Bio-Plex200 (BioRad) according to the manufacturer’s instructions.

### Analysis of Proteomic Data *via* Ingenuity Pathway Analysis (IPA)

Pathway analysis of proteomics data from HL EVs [part of proteomics data were published (16); full list see Table S1 in Supplementary Material] was performed using the IPA tool from QIAGEN (IPA Summer Release 2015, QIAGEN Bioinformatics).

Protein cargo of EVs isolated from the supernatant of the HL cell line KM-H2 was analyzed *via* mass spectroscopy as previously described by our group (16). In brief: the EV proteins were separated with the help of a 10% SDS-PAGE, the lanes subsequently extracted from the gel, reduced (5 mM dithiothreitol, 25 min at 56°C), alkylated (14 mM iodoacetamide, 30 min at RT under exclusion of light), and then digested with trypsin (Promega). Samples were analyzed using an LTQ Velos Orbitrap mass spectrometer (Thermo Fisher Scientific) paired with LC–MS/MS (EASY-nLC system, Proxeon Biosystem). Separated by a 2–90% acetonitrile gradient in 0.1% formic acid, using a PicoFrit Column (20 cm, ID75 μm, 5 µm particle size, New objective) followed. Finally, the full scan MS spectra (*m*/*z* 300–2,000) were checked in the Orbitrap analyzer. Peak lists (msf) were created using Proteome Discoverer version 1.3 (Thermo Fisher Scientific) with a Sequest search engine. The obtained search data were further checked with the software ScaffoldQ + version 3.3.1.

In the context of this study, the results found with the Human Cytokine Array/Chemokine Array 64-Plex Eve Technologies and pre-existing proteomics data (Table S1 in Supplementary Material) were merged in an IPA analysis.

### Mouse Xenograft Model

1 × 10^7^ HDF_n_ cells and 1 × 10^7^ KM-H2 cells were mixed in 100 µl PBS and the cell suspension injected subcutaneously into the lower flank of female NOD scid gamma (NSG) mice (Charles River) to establish tumors. Mice received 50 µg DiO-labeled KM-H2 EVs in 100 µl PBS (*n* = 4) or vehicle control (*n* = 4) *via* intravenous injection into the tail vein at day 2, 4, and 7 after transplantation of tumor cells. Formation of tumors was checked periodically, and tumor volume calculated using the formula (length × width × height)/2. Animals were sacrificed at day 30 and tumor tissue processed for analysis.

### Processing and Histopathology of Tumor Tissue

Upon resection, tumor tissue was snap-frozen in optimal-cutting-temperature compound (Tissue-Tek O.C.T., Sakura Finetek) and sectioned at 5 µm using a HM560 microtome (Thermo Fisher Scientific). Tumor sections were air dried for at least 4 h at RT and stored at −80°C. Hematoxylin and eosin (H&E) staining of cryo-sections was performed for histopathological evaluation of tumor tissue sections were first stained with hematoxylin solution, rinsed with water, and stained with Eosin G solution. Excess dye was cleansed away, the stained sections embedded in GLC Mounting Medium (Sakura Finetek) and sealed with a cover slip. Samples were analyzed with a Keyence Microscope BZ-9000 and the BZ-II Viewer.

### Isolation of Single Cells From Tumor Tissue and Flow Cytometry

Snap-frozen tumor tissue was wet with PBS and kept on ice, chopped with a scalpel and carefully pushed through the mesh of a cell strainer. The cells were re-suspended in 10 ml RPMI 1640 cell culture medium and the suspension overlaid with 20 ml human Ficoll–Paque density gradient medium (GE Healthcare) in a reaction tube. After centrifugation at 2,000 rpm for 20 min at RT (deceleration without brake), the cells were transferred in a fresh tube and washed with PBS for 5 min at 1,200 rpm and pellet resuspended in 1 ml RPMI 1640 and kept on ice. Isolated tumor cells were counted and equal amounts per tumor probed with an APC-conjugated mouse anti-human CD30 antibody (BioLegend, 333909, 1:100) for 30 min on ice and then analyzed *via* flow cytometry as described above.

### Immunohistochemistry

Cryo-sections of tumor tissue were fixed with 1% PFA for 15 min at RT, blocked and permeabilized with 10% normal goat serum and 0.2% Triton X-100 in PBS for 30 min, washed three times with PBS, and subsequently probed with the following primary antibodies: CD30 [clone Ki-4 (17)], CD31 (BD Bioscience, 557355, 1:1,000), and α-SMA conjugated with Cy3 (Sigma-Aldrich, C6198, 1:200). Microscopy slides were incubated with the primary antibody over night at 4°C and then washed in PBS, followed by washing three times with PBS and incubation with the appropriate secondary antibody for 45 min at RT: goat anti-mouse-AF594 (Molecular Probes, A11032, 1:1,000) or goat anti-rat-AF594 (Molecular Probes, A11007, 1:1,000). After washing thrice with PBS, nuclei were stained with DAPI (1 µg/ml). Finally, stained sections were embedded in GLC Mounting Medium and sealed with a cover slip. Samples were analyzed using a Keyence Microscope BZ-9000 and the BZ-II Viewer.

### Statistical Analysis

Experiments were performed independently and at least in three biological replicates, if not stated otherwise. Results obtained from representative experiments are shown. Data are presented as mean + SEM and were analyzed using GraphPad Prism6 software. Statistical significance was calculated as indicated in the figure legends.

### Study Approval

This study was carried out in accordance with § 8 Abs. 1 des Tierschutzgesetzes (animal welfare law of the German Federal Government) and the protocol was approved by the local authorities [Landesamt für Natur, Umwelt und Verbraucherschutz (LANUV), State Northrhine-Westfalia].

## Results

### HL-Derived Vesicles Display Prime Characteristics of EVs

**Figure 1:**
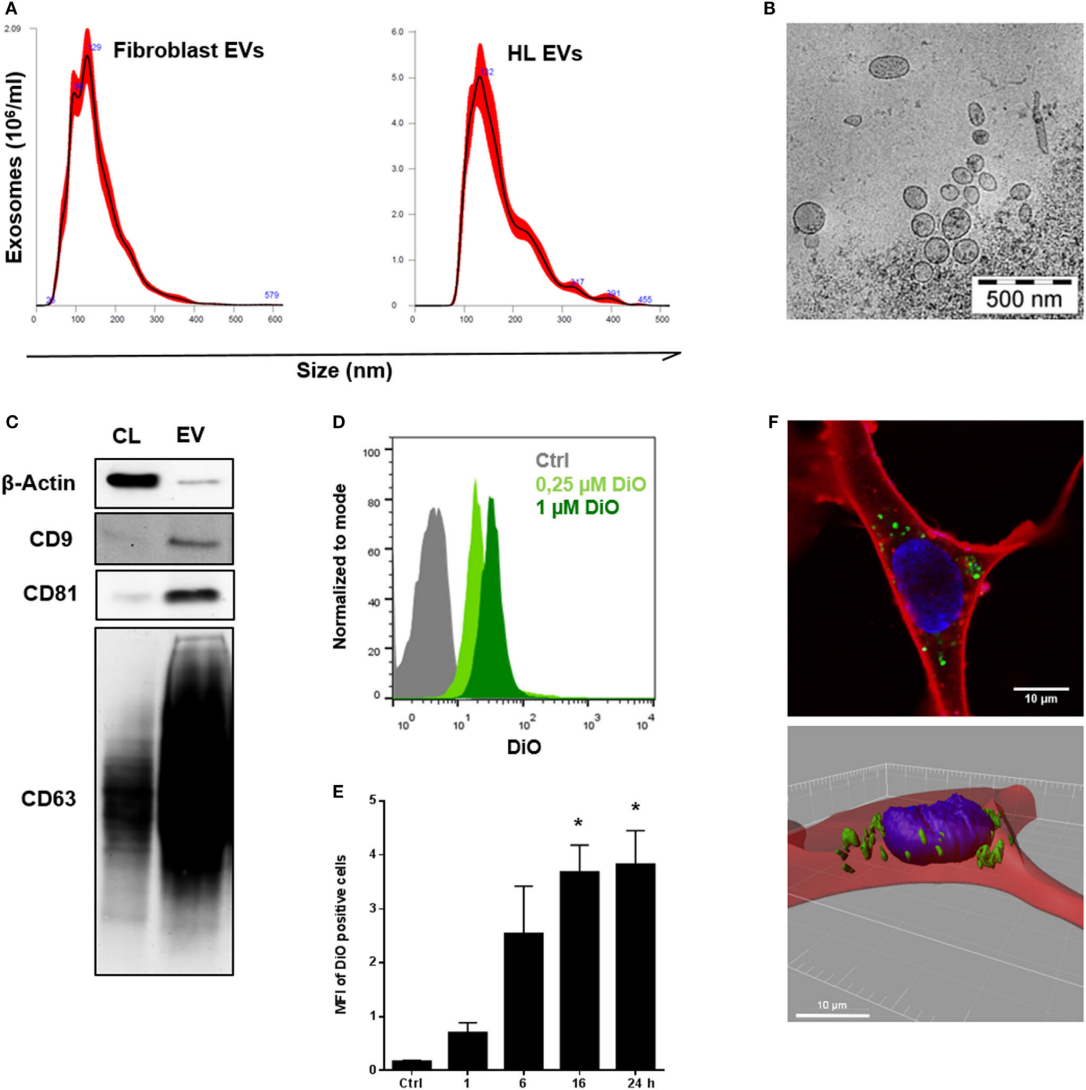
Characterization and internalization of tumor-derived extracellular vesicles (EVs) by fibroblast cells *in vitro*. **(A)** Size distribution of fibroblast EVs (performed once) or Hodgkin lymphoma (HL) EVs (representative experiment, *n* = 3), measured by nanoparticle tracking analysis. **(B)** Electron microscopic image of purified HL EVs (*n* = 1). **(C)** Western blot analysis of exosome markers on HL-derived EVs. Cell lysate of HL cells served as control. Presented is one of three independent experiments. **(D)** Flow cytometry of beads coupled HL EVs which was collected from unstained cells (Ctrl) or from DiO-stained cells (0.25 or 1 µM DiO, *n* = 1). Normalization to mode: events are normalized in a scale with 100 being maximum (FlowJo v10). **(E)** Internalization of DiO-labeled HL EVs by fibroblasts assessed *via* flow cytometry at different time points as indicated. Fibroblast cells were incubated with 100 µg DiO^+^-EVs or DiO-negative EVs as negative control in the presence of EV-depleted medium. Statistical significance was calculated with one-way ANOVA and Tukey’s multiple comparisons test (mean + SEM of three biological replicates; *p ≥ 0.05). **(F)** Visualization of EV-uptake into fibroblasts *via* immunofluorescence: cytoplasm was stained with Cell Mask Deep Red (red), nuclei with Hoechst and HL-derived EVs with DiO (green). Upper picture shows a recipient cell with internalized HL EVs. Bottom picture depicts a three-dimensional view.

Initially, we isolated EVs from the supernatant of healthy fibroblasts and the HL cell line KM-H2 by the means of differential ultracentrifugation (18, 19). NTA of the samples revealed a size distribution of the particles characteristic for small EVs, the so-called exosomes. Representative size distribution plots presented in Figure 1A confirm a mode size of about 130 nm for fibroblast-derived EVs and HL EVs, thus, being in the range typical for EVs obtained from the 100,000 × *g* fraction. The electron microscopy picture shows purified EVs from HL cells (KM-H2) ranging from sizes between 70 and 200 nm (Figure 1B). For further characterization of the EVs, Western blot analysis and flow cytometry were used to identify the proposed markers for EVs of the 100,000 × *g* fraction CD9, CD81, and CD63 (14) on the surface of HL EVs. Western blot analysis confirmed a strong abundance of CD9, CD81, and CD63 on HL EVs (Figure 1C), which could also be confirmed *via* flow cytometry (Figure S1A in Supplementary Material). Moreover, FACS bead assay affirmed the presence of phosphatidylserine and the HL cell marker CD30 on the vesicles, confirming their descent from HL cells (Figure S1B in Supplementary Material).

### Tumor Cell-Derived EVs Are Internalized by Fibroblasts

Next, HL cells were treated with the lipophilic dye DiO (Figure S1C in Supplementary Material). Under these conditions, cells release DiO-stained EVs, which allows to monitor their internalization by fibroblasts. Subsequently, DiO^+^ EVs were purified from cell culture supernatant (Figure 1D). Fibroblasts were challenged with DiO^+^ EVs for different time points as indicated. Flow cytometric analysis revealed a time-dependent binding and/or uptake of DiO^+^ EVs by fibroblasts resulting in DiO-labeling of the recipient cells reaching a maximum after 16 h (Figure 1E). The observed interaction of EVs and fibroblasts was elucidated in more detail by means of confocal microscopy. To this end, fibroblasts were exposed to DiO^+^-EVs for 48 h as described before, but nuclei were stained with Hoechst (blue) and the CellMask Deep Red (red) directly prior to confocal microscopy. Figure 1F (upper picture) shows a representative image of a fibroblast with loaded HL EVs (green). Three-dimensional depiction of the fibroblast from a *z*-axis-series of pictures through the cell (Figure S1D in Supplementary Material) allowed exact determination of the position of internalized DiO^+^-EVs in the analyzed cell (Figure 1F, lower picture). Thus, internalization of HL EVs by fibroblasts was confirmed. However, it is conceivable that even binding of EVs to fibroblasts may contribute to phenotypic changes and signal transduction.

### EVs of HL Cells and Fibroblasts Interact in a Bi-Directional Manner to Enhance Motility and Facilitate Directed Migration

**Figure 2:**
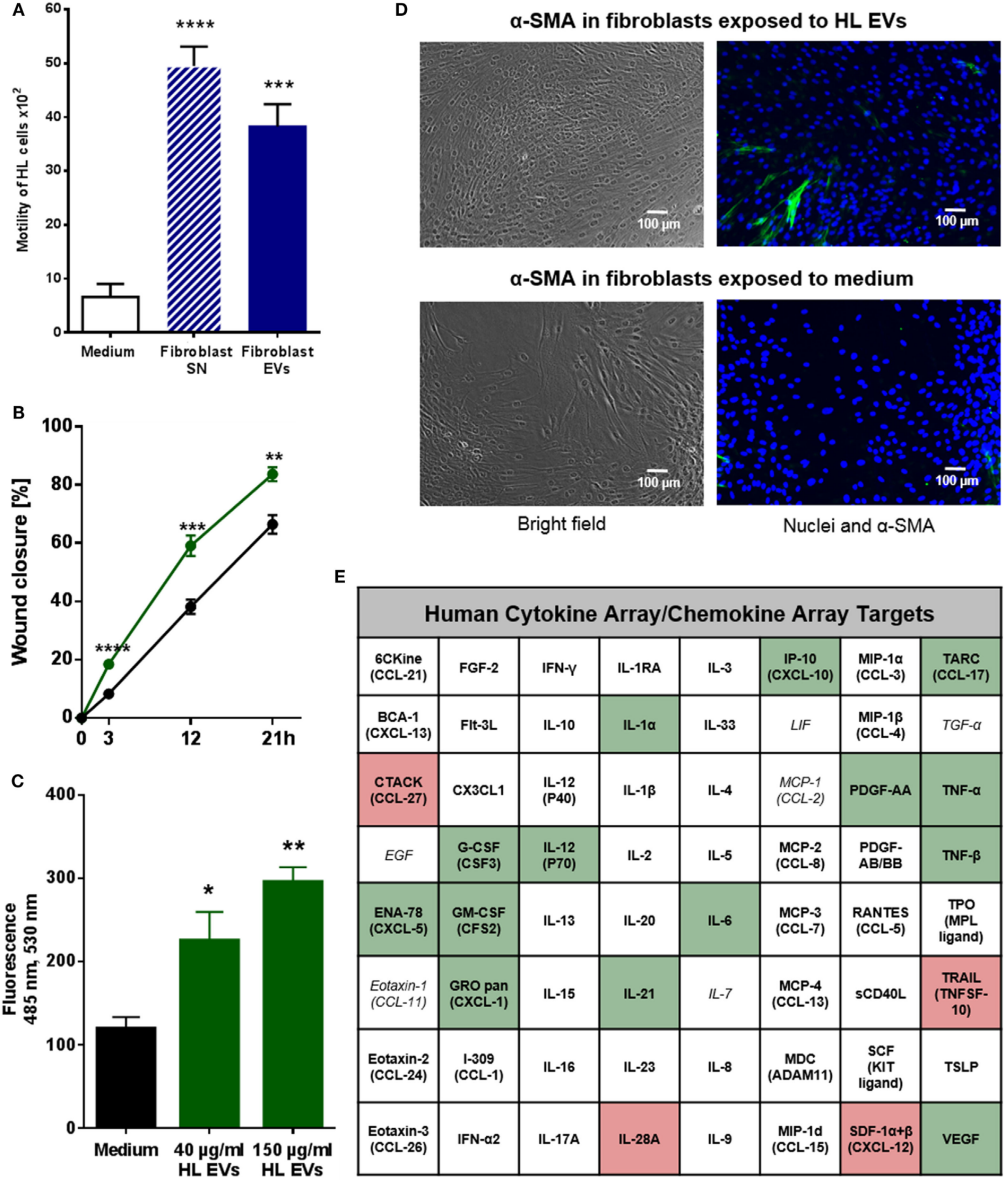
Extracellular vesicles (EVs) promote migration of Hodgkin lymphoma (HL) cells and HL EVs can alter the phenotype of fibroblast cells. **(A)** Transwell migration assay (8 µm pores): HL cells were exposed to supernatant from fibroblast cells or EVs for 26 h. Statistical significance was calculated with one-way ANOVA and Tukey’s multiple comparisons test (presented is mean + SEM, four biological replicates; ***p ≥ 0.001; ****p ≥ 0.0001). **(B)** Scratch assay: 24 h wound closure of fibroblast cells exposed to HL EVs (green) or medium (black). Student’s *t*-test was performed to check for significant differences between both treatments (mean ± SEM, *n* = 3; *p ≥ 0.05; **p ≥ 0.01). **(C)** Chemotaxis assay: migration of labeled fibroblast cells through Neuroprobe ChemoTx plates (5 µm pores) exposed to medium, 40 or 150 µg/ml HL EVs. Statistical assessment was performed with one-way ANOVA and Tukey’s multiple comparisons test (data are presented as mean + SEM, *n* = 4; *p ≥ 0.05; **p ≥ 0.01). **(D)** Exemplary bright field and fluorescence pictures of a scratch assay after 24 h exposure of fibroblasts to 100 µg/ml (≈1 × 10^9^ EVs/ml) HL EVs or medium. Nuclei stained with DAPI (blue), fibroblast cells with α-SMA (green). This experiment was performed in three independent replicates. **(E)** Human 64-Plex chemokine array: quantification of chemokines/cytokines after 24 h in the supernatant of fibroblasts in presence or absence of HL EVs. Not detected cytokines/chemokines are depicted in italic letters; abundant factors are in bold; enhanced expression is highlighted in green, whereas red boxes indicate lower expression after exposure to HL EVs. Statistical differences in abundance of chemokines/cytokine were determined using Student’s *t*-test (three independent replicates).

Within the TME, both malignant and non-malignant cells interact to prepare a favorable surrounding for the tumor. We aimed to dissect the role of vesicular factors in these bi-directional communication processes with main focus on the impact of EVs from HL cells on the motility and migration of fibroblasts and *vice versa*. Initially, we measured the proliferation of HDF_n_ cells co-cultivated with HL EVs and conversely KM-H2 cells co-cultivated with HDF_n_ EVs. Results of the XTT assays did not show any relevant differences in cell proliferation in presence or absence of EVs for both tested cell lines (Figures S3C,D in Supplementary Material) suggesting that the proliferation is not affected by internalized EVs. Next, we studied the role of fibroblast-derived EVs using a transwell approach. Of note, the mobility of HL cells was significantly increased by crude fibroblast supernatant, containing all secreted molecules and vesicles, and by purified fibroblast EVs, compared to control cells incubated with medium (Figure 2A). The mobility of HL cells was slightly higher, but not significantly increased, after incubation with the supernatant in comparison to purified fibroblast EVs, implying that also soluble factors in the supernatant might influence tumor cell mobility. The bi-directional cross-talk of HL EVs on fibroblasts was investigated by means of the so-called scratch assay in which the wound closure of a fibroblast cell monolayer was tested in presence or absence of HL EVs (21 h). The experiment confirms a significant enhancement of wound closure/directed migration of fibroblasts in response to HL EVs (Figure 2B). Chemotaxis plays a central role in directed migration of cells. To this end, the influence of HL EVs on the directed migration of fibroblasts was tested with Neuroprobe ChemoTx plates in which cells can migrate through a membrane toward an attractant. Statistical evaluation in Figure 2C confirms a dose-dependent attraction of fibroblasts by HL EVs. In conclusion, we demonstrated that the bi-directional communication of HL cells and stromal cells/fibroblasts *via* EVs impacts on the motility of HL cells and facilitates the directed migration of fibroblasts.

### HL-Derived EVs Promote Transition of Healthy Fibroblasts to a Cancer-Associated Phenotype

Given the evidence for internalization of HL EVs by fibroblasts and their positive effect on the migration, we set out to investigate the effects of HL EVs on the phenotype of fibroblasts. Therefore, we combined the scratch assay as described before with immunofluorescence to evaluate the abundance of alpha-smooth muscle actin protein (α-SMA), a commonly used marker for cancer-associated fibroblasts (CAFs) (20). Representative pictures in Figure 2D indicate a higher number of α-SMA positive cells upon exposure to HL EVs, pointing to their activation toward a CAF phenotype. Of note, treatment with TGF-β as a positive control (Figure S2A in Supplementary Material, lower panel) did not provoke a higher ratio of α-SMA positive fibroblasts. This might reflect the herterogenicity of the HDF_n_ cells since α-SMA is, besides being expressed in CAFs, the most significant marker for myofibroblasts (21). This observation is in line with the work of Koumas and colleagues in which TGF-β induced expression of α-SMA was observed in a part of the assessed fibroblast population only (22).

Transition of fibroblasts into a CAF phenotype is characterized by alterations in gene expression affecting different cellular pathways including the cell’s secretome (20), which was further studied using a 64-Plex Chemokine Array. Fibroblasts were exposed to HL EVs or medium for 24 h and their secretome in the presence or absence of EVs was analyzed. 19 of the 64 assayed chemokines/cytokines showed significantly altered levels in the supernatant of fibroblasts treated with HL EVs demonstrating that the phenotypical change correlates with modulation of the release of soluble factors (Figure 2E; Figure S2 in Supplementary Material). Of note, secretion of the chemokines and cytokines is known as a critical factor in HL pathogenesis (23) and disease relevant molecules such as TNF-α are enhanced in presence of HL EVs with highest significance. Besides of that, a group of chemotactic cytokines (e.g., ENA-78, GRO pan) were measurable in higher levels as well as the pro-inflammatory NF-κB-induced cytokines IL-1α and IL-6. This was in line with a higher abundance of growth factors (G-CSF, GM-CSF) and angiogenesis stimulating factors (VEGF). All of these cytokines/chemokines are important for the establishment of a tumor-favorable environment in HL. Strikingly, most of the altered targets are part of the signaling network of the inflammatory master key regulator NF-κB, a driver pathway in HL (4, 24, 25). In line, IPA (Qiagen) of HL-EV proteomics data from our previous studies (16) (Table S1 in Supplementary Material) merged with the results from the Cytokine array (Figure 2E; Figure S2B in Supplementary Material) revealed inflammatory pathways, including TNF-α/NF-κB-signaling, as key factors directing the EV-dependent phenotype changes in fibroblasts. The core analysis of altered cytokines/chemokines unraveled a key role for the inflammatory mediators STAT3, IFNγ, TNF-α and the involvement of TOLL-like receptor-, reactive oxygen species-, and NF-κB-signaling (Figure S4 in Supplementary Material).

*Vice versa*, ROS signaling and NF-κB-signaling pathways popped up upon IPA of the proteins identified by mass spectrometry of HL EV protein cargo isolated from the supernatant of KM-H2 cells. These results point to a central role of the TNF-α/NF-κB axis in HL EV-mediated alteration in recipient fibroblast.

### HL EVs Promote a CAF Phenotype and Vascularization in a Xenograft Model

**Figure 3:**
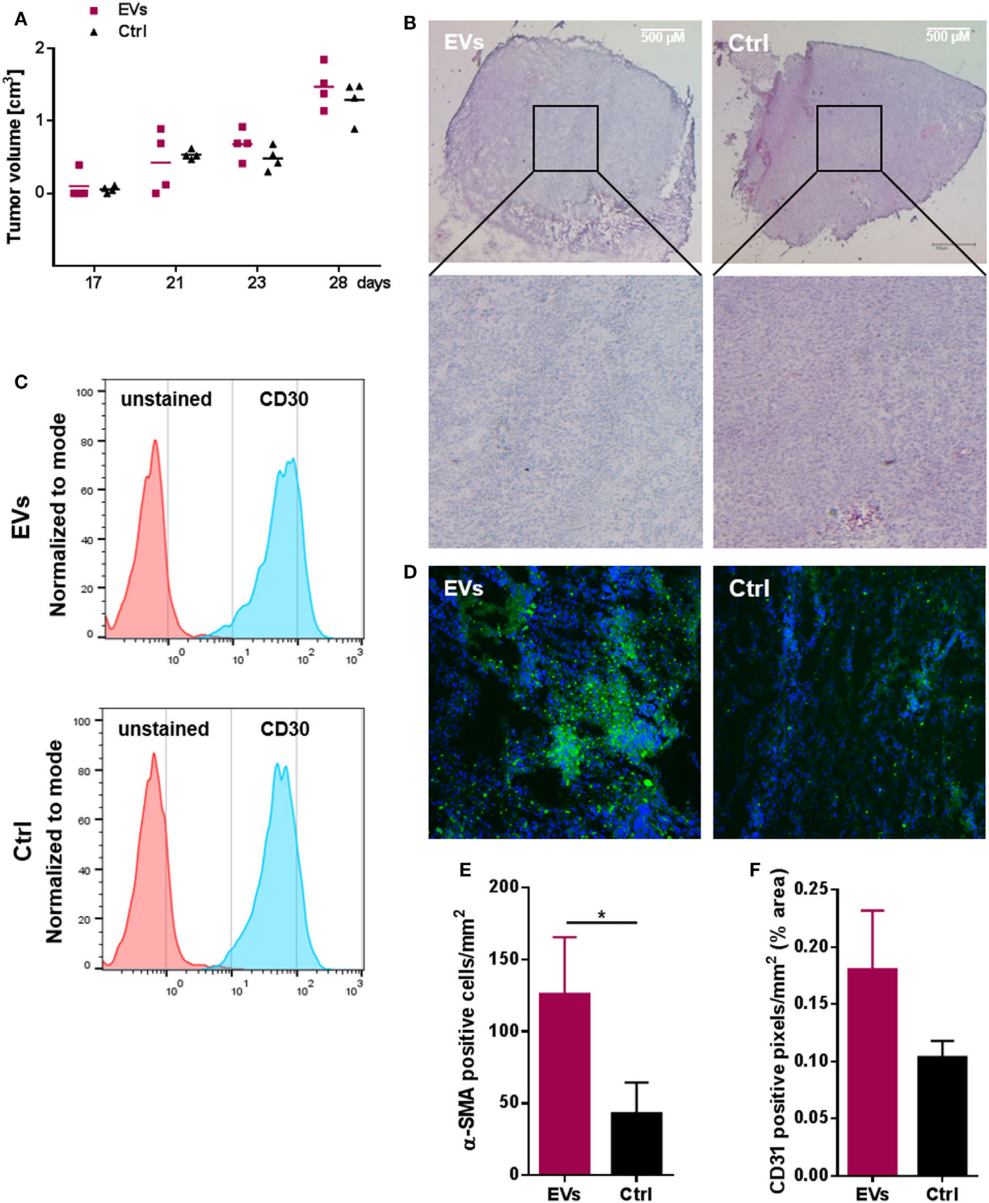
Hodgkin lymphoma (HL) xenograft model. 1 × 10^7^ HDF_n_ + 1 × 10^7^ KM-H2 cells were subcutaneously transplanted into NOD scid gamma mice (age 141 days). Treatment group (*n* = 4 animals) received an i.v. injection of HL extracellular vesicles (EVs) administered *via* the tail vein at day 22, 25, 27, and 28, animals of the control group (*n* = 4 animals) were injected with PBS. Necropsy was performed on day 30. **(A)** Tumor growth in EV-treated and control animals; tumor volume was assessed as tumors were detectable (*n* = 4 animals per group). **(B)** Representative hematoxylin and eosin stainings of tumor tissue cryo-sections (25× magnification) from EV-treated (EVs) or control (Ctrl) animals (four animals per group were analyzed). **(C)** Abundance of CD30 on cells of four resected tumors analyzed by flow cytometry. **(D)** DiO-positive tumor cells after internalization of labeled HL EVs (*n* = 4). **(E)** Number of α-SMA positive cells and **(F)** cells expressing the vascularization marker CD31 in tumor sections of both groups assessed microscopically and quantified using ImageJ software (http://rsb.info.nih.gov/ij) (Student’s *t*-test of *n* ≥ 3 samples presented as mean + SEM; *p ≥ 0.05).

To investigate the *in vivo* impact of HL EVs on fibroblasts, we applied a HL xenograft model with KM-H2 cells to immunodeficient NSG mice. Fibroblasts and HL cells (1:1) were subcutaneously transplanted into the lower flank of NSG mice (age 141 days). Animals of the treatment group received an intravenous injection of 50 µg DiO^+^ HL EVs at day 22, 25, 27, and 28, animals of the control group were injected with PBS. Necropsy was performed at the end of the experiment and tumor growth was monitored as soon as tumors were visible. Both groups showed a comparable tumor growth/volume in this model (Figure 3A). Figure 3B shows representative overview and detail pictures of tumor sections stained with H&E from EV-treated mice and control animals. In accordance with the comparable tumor growth, tumors of both groups showed a similar histology and similar staining of nuclei (blue), cytoplasm and the extracellular matrix (pink). Flow cytometry revealed a high abundance of CD30-positive cells in tumor tissue of EV-treated and control animals (Figure 3C). After we successfully confirmed the growth of human HL cells in immunodeficient NSG mice, the effect of HL-derived EVs on the migration of fibroblasts and the induction of a CAF phenotype was investigated. Figure 3E shows statistical evaluation of α-SMA levels in tumor tissue sections (Figure S3A in Supplementary Material) of both groups with the number of α-SMA-positive fibroblasts being 2.93-fold higher in the EV-treated group. Moreover, the angiogenesis marker CD31 was increased in the tumor tissue of EV-treated animals pointing to a higher vascularization (1.74-fold increase) in tumors of EV-treated mice (Figure 3F; Figure S3B in Supplementary Material). Of note, we could observe the DiO^+^ HL EVs in sections of paraffin-embedded tumor tissue *via* immunofluorescence (Figure 3D). Collectively, we successfully established human HL tumors in immunodeficient NSG mice and, moreover, found evidence for alteration of the phenotypes of fibroblasts in the TME toward an activated CAF phenotype. Histological analysis of tumor tissue revealed higher vascularization in tumor tissue caused by the administered HL-derived EVs.

Altogether, HL EVs have shown to modulate cellular activities and are able to re-program the phenotype in fibroblasts promoting a suitable TME for tumor growth and progression.

## Discussion

Bi-directional communication between malignant cells and the cells composing the TME is critical for tumor growth, progression, and metastasis. This is of particular importance in HL since few malignant cells interact with a large number of stroma cells to establish a tumor-supportive environment (7).

Here, we provide evidence that HL cell-derived EVs modulate the TME by re-programming or educating fibroblasts to promote a tumor supporting environment.

This conclusion is based on the observation that fibroblast internalize HL EVs (1) causing an increased migration capacity of the recipient cells (2), which was associated with the induced release of cytokines/chemokines relevant for HL tumor progression (3).

Fibroblasts found in association with HL cells (so-called HL-AF for HL-activated fibroblasts) (7) release growth factors and cytokines, such as TGF-β or IL-6 into the surrounding malignant tissue to support tumor growth and maintenance (26). However, the mechanisms underlying the transformation from healthy fibroblasts to HL-AF are not fully understood (27). One of the factors involved is IL-7 released by HL cells which triggers IL-6 production in fibroblasts (28). Moreover, HL cells release IL-13, TNF-α, and TGF-β thereby promoting fibroblast proliferation (29).

In this work, we present first evidence that tumor cell-derived EVs are also able to shape the phenotype of fibroblasts. A contribution of both soluble and vesicular components was demonstrated: the crude cell supernatant, the soluble fraction, and purified vesicles were able to educate fibroblasts toward a tumor-promoting phenotype. These findings complement data demonstrating that the EV-dependent cell-cell communication between distant cells in HL involves CD30-expressing HL EVs. CD30 is a receptor of the TNF receptor superfamily and responsible for constitutive NF-κB-signaling in HL cells, which contributes to HL pathogenesis. It was shown that CD30-HL EVs are guided by a network of protrusions to CD30L-positive granulocytes and neutrophils to induce the release of IL-8, which triggers angiogenesis (16). In line with our findings, Giannoni and colleagues reported a crucial role of carcinoma-derived vesicular IL-6 in the activation of fibroblasts (30). Vesicular activators of the NF-κB-signaling pathway (e.g., TNF-α and TGF-β) secreted by prostate cancer cells trigger the differentiation of fibroblasts into CAFs, promote stemness, and angiogesis (31, 32). These data suggest that EVs play a fundamental role in the organization of the TME.

Factors that were released by fibroblasts in response to EV-treatment include numerous molecules that shape the TME and the associated non-malignant cells (Figure S2 in Supplementary Material). Among these is TARC, a chemokine which binds to the chemokine receptor CCR4 expressed on malignant cells, regulatory T cells, and Th2 cells that are enriched in tumor tissue. Thus, TARC promotes the inflammatory HL TME (33). Of note, enhanced TARC serum levels correlate with a bad prognosis for HL patients and is proposed as a possible biomarker for disease (10). Furthermore, secretion of growth factors (G-CSF, GM-CSF) and angiogenesis stimulating factors (VEGF), which is induced in EV-treated fibroblasts, is known to promote a tumor-supportive environment in hematological and solid tumors (34–37).

In line with this study, it was reported that EVs collected from chronic lymphocytic leukemia (CLL) transfer their molecular cargo to stromal cells to induce a phenotype corresponding to CAFs resulting in increased angiogenesis and the release of pro-survival chemokines/cytokines (38). The molecular basis of these pro-inflammatory, tumor supporting EV-mediated activation is only partly defined. One critical factor seems to be the protein S100-A9 which activates the NF-κB pathway during CLL progression in CLL cells in an autocrine loop (39). Another recent study showed that tumor cell-derived EVs are able to trigger TGF-β-dependent fibroblast-differentiation toward a phenotype which supports angiogenesis and tumor growth (40). However, the molecular basis for the activity of EVs which shape the TME is complex and remains to be investigated in more detail. Mass spectrometry of HL EVs isolated from the supernatant of KM-H2 cells revealed mTOR-signaling, protein ubiquitination, ROS signaling and NF-κB-signaling as prominent canonical pathways. These results suggest the involvement of TNF-α/NF-κB pathways in the functionality of HL EV, and this is one of the most relevant tumor drivers involved in the pathobiology of Hodgkin’s disease (3, 41).

A relevant and immunocompetent mouse model for HL is not available; hence, we used a xenograft model to confirm that HL EVs can modulate the tumor microenvironment. Treatment of animals with HL EVs after transplantation of HL cells and fibroblast cells did not influence tumor growth compared to the control group, which did not receive EVs. Potential growth differences are probably not detectable in this fast-developing tumor model, at least in the absence of an immune system. However, the induction of the CAF marker α-SMA could be observed in tumor tissue of animals receiving HL EVs and this was associated with higher blood vessel formation. Of note, we could detect DiO^+^ HL EVs in sections of paraffin-embedded tumor tissue *via* immunofluorescence indicating an accumulation of tumor-derived vesicles at the tumor site.

**Figure 4:**
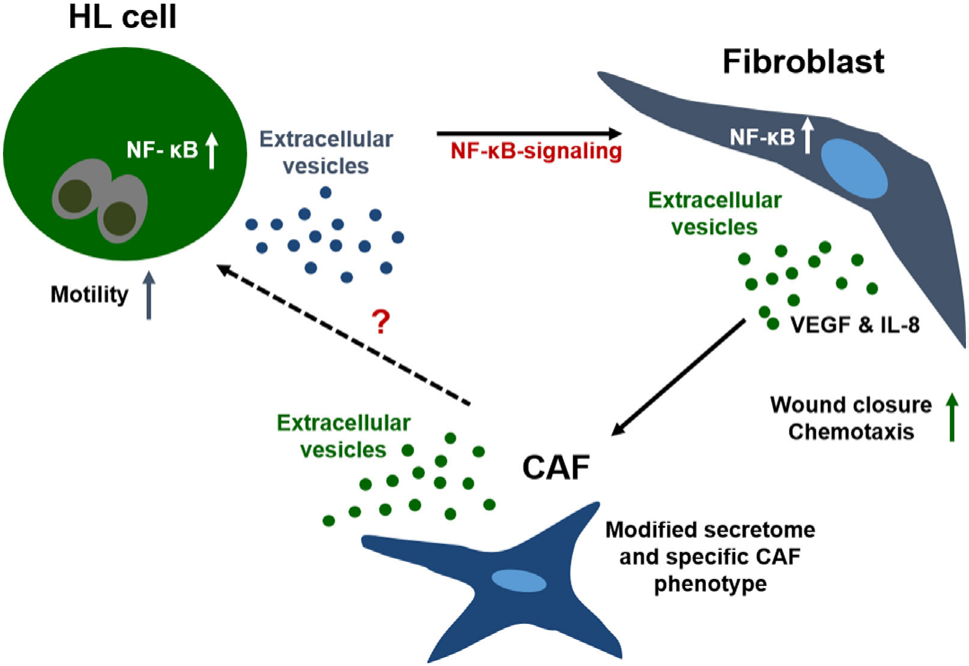
Interaction of fibroblasts and Hodgkin lymphoma (HL) cells in the tumor microenvironment. Communication of the few malignant cells in HL with fibroblasts is crucial for survival of the tumor cells. Due to the spatial distance between the cells, direct cell–cell interaction is not the first-line communication mechanism. Interaction of both cell types is facilitated by soluble and vesicular factors, e.g., chemokines/cytokines, which enhance motility and migration of cells. Further, HL extracellular vesicles shape the phenotype of fibroblasts, skewing the cells to a cancer-associated cell state concomitant with alteration of their secretome. Our results suggest a strong involvement of the TNF-α/NF-κB axis in this process.

Taken together, we provide evidence for a model of bi-directional cross-talk *via* EVs and soluble factors between HL cells and non-malignant stromal cells both *in vitro* and *in vivo*. Within this network, HL EVs shape the phenotype of fibroblasts, skewing their phenotype to a cancer-associated cell state and leading to changes in the secretome of fibroblasts (Figure 4). We propose that a deregulated NF-κB pathway in HRS cells critically contributes to HL EV function, since a NF-κB signature was identified in HL EV samples, using IPA. Alteration of NF-κB-signaling pathways in fibroblasts mediated by signal molecules in HL EV-cargo should be addressed in future studies in more detail. In this study, we identified potential players including IL-1α, IL-6, TNF-α, and VEGF. A better understanding of the complex interactions in HL and the extended knowledge about the role of EVs in that context might aid to develop novel therapeutic tools to fight cancer.

## Ethics Statement

This study was carried out in accordance with § 8 Abs. 1 des Tierschutzgesetzes and the protocol was approved by the local authorities [Landesamt für Natur, Umwelt und Verbraucherschutz (LANUV), State Northrhine-Westfalia].

## Author Contributions

ES, HH, and AE: designed research and analyzed data. TB, KR, SB, RS, OS, and PZ: performed research. ES, BD, and KR wrote the paper.

## Conflict of Interest Statement

The authors declare that the research was conducted in the absence of any commercial or financial relationships that could be construed as a potential conflict of interest.

## Supplementary Material

Figure S1 **(A)** Representative histograms of flow cytometric analysis of exosome markers **(B)** as well as phosphatidylserine and the Hodgkin lymphoma (HL) marker CD30 on HL-derived extracellular vesicles (EVs) (line) compared to isotype control (solid blot). The in **(A,B)** presented FACS analysis was performed in three independent experiments. **(C)** Flow cytometric evaluation of HL cells stained with 0.25 or 1 µm DiO compared to unstained cells (Ctrl). **(D)** EV-uptake in fibroblasts cells studied *via* IF: cytoplasm was stained with cell mask deep red (red), nuclei with Hoechst and HL-derived EVs with DiO (green). Depicted are three representative pictures from upper, middle, and bottom section of a fibroblast.

Figure S2 **(A)** Representative bright field and fluorescence pictures of a scratch assay after 24 h exposure of fibroblasts to 100 µg/ml Hodgkin lymphoma (HL) extracellular vesicles (EVs), medium or 5 ng/ml TGF-β as a positive control. Nuclei are depicted in blue and abundance of α-SMA in green. Microscopic assessment of α-SMA was performed for all three independent scratch assays summarized in Figure 2B. **(B)** Statistical evaluation of the Human 64-Plex Chemokine Array. Abundance of chemokines/cytokines after 24 h in the supernatant of fibroblasts under influence of HL EVs compared to cells incubated with normal medium (white bars: decrease, black bars: increase). Statistical differences were determined with a Student’s *t*-test (mean + SEM of three independent replicates; **p* ≥ 0.05; ***p* ≥ 0.01; ****p* ≥ 0.001; *****p* ≥ 0.0001).

Figure S3 Representative pictures of tumor tissue sections of EV-treated animals and control animals stained for **(A)** α-SMA or **(B)** CD31 (red), nuclei were stained with DAPI (blue). Staining was performed for four animals per group. **(C)** Cell proliferation (XTT assay) of HDF_n_ cells treated with 100 or 200 µg/ml KM-H2 extracellular vesicles (EVs) for the indicated time periods and **(D)** KM-H2 cells exposed to 100 or 200 µg/ml HDF_n_ EVs over the indicated time course. Depicted is one experiment per cell line. The red numbers display a decrease of cell proliferation compared to the PBS-treated control, whereas green numbers express an increase of proliferation after exposure to EVs. Absorbance was measured at 475 nm with 660 nm as reference wave length.

Figure S4 Pathway analysis of proteomics data obtained from analysis of Hodgkin lymphoma extracellular vesicles using the ingenuity pathway analysis (IPA) tool from QIAGEN (IPA Summer Release 2015, QIAGEN Bioinformatics).

Video S1 Scratch assay to monitor the migration of fibroblasts in presence of HL EVs.

Video S2 Scratch assay to monitor the migration of fibroblasts in absence of HL EVs.

## References

[1] Hodgkin T On some morbid appearances of the absorbent glands and spleen. Med Chir Trans (1832) 17:68–114. 10.1177/095952873201700106 PMC2116706 20895597

[2] DeVita VT Costa J Toward a personalized treatment of Hodgkin’s disease. N Engl J Med (2010) 362:942–3. 10.1056/NEJMe0912481 20220189

[3] Küppers R Engert A Hansmann M-L. Hodgkin lymphoma. J Clin Invest (2012) 122:3439–47. 10.1172/JCI61245 23023715 PMC3534167

[4] Aldinucci D Gloghini A Pinto A de Filippi R Carbone A. The classical Hodgkin’s lymphoma microenvironment and its role in promoting tumour growth and immune escape. J Pathol (2010) 221:248–63. 10.1002/path.2711 20527019

[5] Tzankov A Zimpfer A Pehrs A-C Lugli A Went P Maurer R Expression of B-cell markers in classical Hodgkin lymphoma: a tissue microarray analysis of 330 cases. Mod Pathol (2003) 16:1141–7. 10.1097/01.MP.0000093627.51090.3F 14614054

[6] Steidl C Connors JM Gascoyne RD. Molecular pathogenesis of Hodgkin’s lymphoma: increasing evidence of the importance of the microenvironment. J Clin Oncol (2011) 29:1812–26. 10.1200/JCO.2010.32.8401 21483001

[7] Aldinucci D Celegato M Casagrande N. Microenvironmental interactions in classical Hodgkin lymphoma and their role in promoting tumor growth, immune escape and drug resistance. Cancer Lett (2016) 380:243–52. 10.1016/j.canlet.2015.10.007 26474544

[8] Zocchi MR Catellani S Canevali P Tavella S Garuti A Villaggio B High ERp5/ADAM10 expression in lymph node microenvironment and impaired NKG2D ligands recognition in Hodgkin lymphomas. Blood (2012) 119:1479–89. 10.1182/blood-2011-07-370841 22167753

[9] Reiners KS Topolar D Henke A Simhadri VR Kessler J Sauer M Soluble ligands for NK cell receptors promote evasion of chronic lymphocytic leukemia cells from NK cell anti-tumor activity. Blood (2013) 121:3658–65. 10.1182/blood-2013-01-476606 23509156 PMC3643764

[10] Sauer M Plütschow A Jachimowicz RD Kleefisch D Reiners KS Ponader S Baseline serum TARC levels predict therapy outcome in patients with Hodgkin lymphoma. Am J Hematol (2013) 88:113–5. 10.1002/ajh.23361 23225085

[11] Zocchi MR Camodeca C Nuti E Rossello A Venè R Tosetti F ADAM10 new selective inhibitors reduce NKG2D ligand release sensitizing Hodgkin lymphoma cells to NKG2D-mediated killing. Oncoimmunology (2016) 5:e1123367 10.1080/2162402X.2015.1123367 27467923 PMC4910733

[12] Ruivo CF Adem B Silva M Melo SA The biology of cancer exosomes: insights and new perspectives. Cancer Res (2017) 77(23):6480–8. 10.1158/0008-5472.CAN-17-0994 29162616

[13] Dörsam B Reiners KS von Strandmann EP. Cancer-derived extracellular vesicles: friend and foe of tumour immunosurveillance. Philos Trans R Soc Lond B Biol Sci (2018) 373. 10.1098/rstb.2016.0481 29158311 PMC5717436

[14] Kowal J Arras G Colombo M Jouve M Morath JP Primdal-Bengtson B Proteomic comparison defines novel markers to characterize heterogeneous populations of extracellular vesicle subtypes. Proc Natl Acad Sci U S A (2016) 113:E968–77. 10.1073/pnas.1521230113 26858453 PMC4776515

[15] Simhadri VR Reiners KS Hansen HP Topolar D Simhadri VL Nohroudi K Dendritic cells release HLA-B-associated transcript-3 positive exosomes to regulate natural killer function. PLoS One (2008) 3:e3377. 10.1371/journal.pone.0003377 18852879 PMC2566590

[16] Hansen HP Engels H-M Dams M Paes Leme AF Pauletti BA Simhadri VL Protrusion-guided extracellular vesicles mediate CD30 trans-signalling in the microenvironment of Hodgkin’s lymphoma. J Pathol (2014) 232:405–14. 10.1002/path.4306 24659185

[17] Horn-Lohrens O Tiemann M Lange H Kobarg J Hafner M Hansen H Shedding of the soluble form of CD30 from the Hodgkin-analogous cell line L540 is strongly inhibited by a new CD30-specific antibody (Ki-4). Int J Cancer (1995) 60:539–44. 10.1002/ijc.2910600419 7530238

[18] Théry C Amigorena S Raposo G Clayton A. Isolation and characterization of exosomes from cell culture supernatants and biological fluids. Curr Protoc Cell Biol (2006) Chapter 3:Unit3.22. 10.1002/0471143030.cb0322s30 18228490

[19] Livshits MA Livshts MA Khomyakova E Evtushenko EG Lazarev VN Kulemin NA Isolation of exosomes by differential centrifugation: theoretical analysis of a commonly used protocol. Sci Rep (2015) 5:17319. 10.1038/srep17319 26616523 PMC4663484

[20] Augsten M. Cancer-associated fibroblasts as another polarized cell type of the tumor microenvironment. Front Oncol (2014) 4:820. 10.3389/fonc.2014.00062 24734219 PMC3973916

[21] Goldberg MT Han Y-P Yan C Shaw MC Garner WL. TNF-alpha suppresses alpha-smooth muscle actin expression in human dermal fibroblasts: an implication for abnormal wound healing. J Invest Dermatol (2007) 127:2645–55. 10.1038/sj.jid.5700890 17554369 PMC2366884

[22] Koumas L Smith TJ Feldon S Blumberg N Phipps RP. Thy-1 expression in human fibroblast subsets defines myofibroblastic or lipofibroblastic phenotypes. Am J Pathol (2003) 163:1291–300. 10.1016/S0002-9440(10)63488-8 14507638 PMC1868289

[23] Hsu PL Hsu SM. Production of tumor necrosis factor-alpha and lymphotoxin by cells of Hodgkin’s neoplastic cell lines HDLM-1 and KM-H2. Am J Pathol (1989) 135:735–45. 2801887 PMC1880038

[24] Jost PJ Ruland J. Aberrant NF-kappaB signaling in lymphoma: mechanisms, consequences, and therapeutic implications. Blood (2007) 109:2700–7. 10.1182/blood-2006-07-025809 17119127

[25] Weniger MA Küppers R NF-κB deregulation in Hodgkin lymphoma. Semin Cancer Biol (2016) 39:32–9. 10.1016/j.semcancer.2016.05.001 27221964

[26] Jia C-C Wang T-T Liu W Fu B-S Hua X Wang G-Y Cancer-associated fibroblasts from hepatocellular carcinoma promote malignant cell proliferation by HGF secretion. PLoS One (2013) 8:e63243. 10.1371/journal.pone.0063243 23667593 PMC3647063

[27] Kalluri R Zeisberg M Fibroblasts in cancer. Nat Rev Cancer (2006) 6:392–401. 10.1038/nrc1877 16572188

[28] Cattaruzza L Gloghini A Olivo K Di Francia R Lorenzon D de Filippi R Functional coexpression of interleukin (IL)-7 and its receptor (IL-7R) on Hodgkin and Reed-Sternberg cells: involvement of IL-7 in tumor cell growth and microenvironmental interactions of Hodgkin’s lymphoma. Int J Cancer (2009) 125:1092–101. 10.1002/ijc.24389 19391137

[29] Aldinucci D Lorenzon D Olivo K Rapanà B Gattei V. Interactions between tissue fibroblasts in lymph nodes and Hodgkin/Reed-Sternberg cells. Leuk Lymphoma (2004) 45:1731–9. 10.1080/10428190410001683633 15223630

[30] Giannoni E Bianchini F Masieri L Serni S Torre E Calorini L Reciprocal activation of prostate cancer cells and cancer-associated fibroblasts stimulates epithelial-mesenchymal transition and cancer stemness. Cancer Res (2010) 70:6945–56. 10.1158/0008-5472.CAN-10-0785 20699369

[31] Fiaschi T Giannoni E Taddei ML Cirri P Marini A Pintus G Carbonic anhydrase IX from cancer-associated fibroblasts drives epithelial-mesenchymal transition in prostate carcinoma cells. Cell Cycle (2013) 12:1791–801. 10.4161/cc.24902 23656776 PMC3713137

[32] Ramteke A Ting H Agarwal C Mateen S Somasagara R Hussain A Exosomes secreted under hypoxia enhance invasiveness and stemness of prostate cancer cells by targeting adherens junction molecules. Mol Carcinog (2015) 54:554–65. 10.1002/mc.22124 24347249 PMC4706761

[33] Küppers R. The biology of Hodgkin’s lymphoma. Nat Rev Cancer (2009) 9:15–27. 10.1038/nrc2542 19078975

[34] Aguayo A Kantarjian H Manshouri T Gidel C Estey E Thomas D Angiogenesis in acute and chronic leukemias and myelodysplastic syndromes. Blood (2000) 96:2240–5. 10979972

[35] Peinado H Alečković M Lavotshkin S Matei I Costa-Silva B Moreno-Bueno G Melanoma exosomes educate bone marrow progenitor cells toward a pro-metastatic phenotype through MET. Nat Med (2012) 18:883–91. 10.1038/nm.2753 22635005 PMC3645291

[36] Costa-Silva B Aiello NM Ocean AJ Singh S Zhang H Thakur BK Pancreatic cancer exosomes initiate pre-metastatic niche formation in the liver. Nat Cell Biol (2015) 17:816–26. 10.1038/ncb3169 25985394 PMC5769922

[37] Yang J Li W He X Zhang G Yue L Chai Y. VEGF overexpression is a valuable prognostic factor for non-Hodgkin’s lymphoma evidence from a systemic meta-analysis. Dis Markers (2015) 2015:786790. 10.1155/2015/786790 25810565 PMC4355555

[38] Paggetti J Haderk F Seiffert M Janji B Distler U Ammerlaan W Exosomes released by chronic lymphocytic leukemia cells induce the transition of stromal cells into cancer-associated fibroblasts. Blood (2015) 126:1106–17. 10.1182/blood-2014-12-618025 26100252 PMC4560344

[39] Prieto D Sotelo N Seija N Sernbo S Abreu C Durán R S100-A9 protein in exosomes from chronic lymphocytic leukemia cells promotes NF-κB activity during disease progression. Blood (2017) 130:777–88. 10.1182/blood-2017-02-769851 28596424

[40] Webber JP Spary LK Sanders AJ Chowdhury R Jiang WG Steadman R Differentiation of tumour-promoting stromal myofibroblasts by cancer exosomes. Oncogene (2015) 34:290–302. 10.1038/onc.2013.560 24441045

[41] Jundt F Anagnostopoulos I Bommert K Emmerich F Müller G Foss HD Hodgkin/Reed-Sternberg cells induce fibroblasts to secrete eotaxin, a potent chemoattractant for T cells and eosinophils. Blood (1999) 94:2065–71. 10477736

