# Supplementary figures and images for: Hodgkin Lymphoma-Derived Extracellular Vesicles Change the Secretome of Fibroblasts Toward a CAF Phenotype
Source: Front Immunol. 2018 Jun 18;9:1358. doi: 10.3389/fimmu.2018.01358 (PMC6015880; doi:10.3389/fimmu.2018.01358)

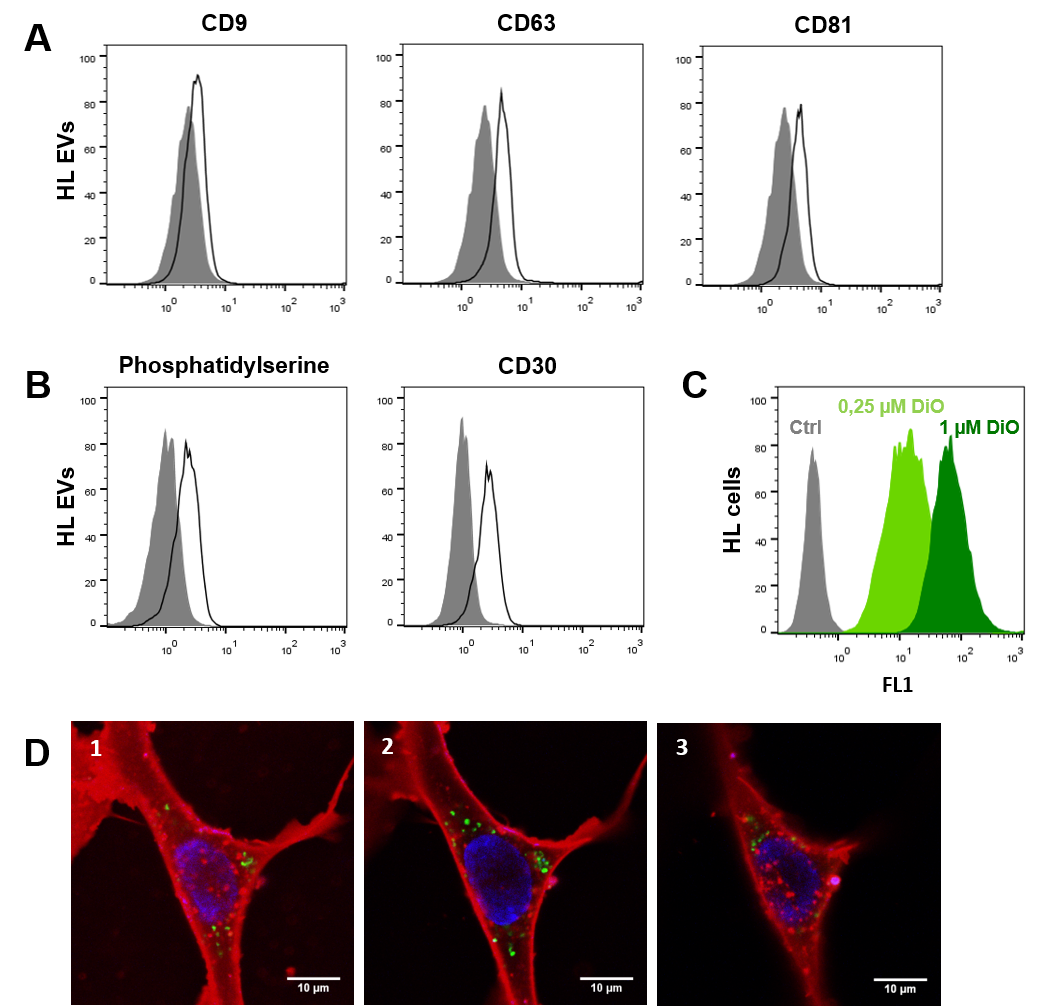

Supplement: Figure S1 — (A) Representative histograms of flow cytometric analysis of exosome markers (B) as well as phosphatidylserine and the Hodgkin lymphoma (HL) marker CD30 on HL-derived extracellular vesicles (EVs) (line) compared to isotype control (solid blot). The in (A,B) presented FACS analysis was performed in three independent experiments. (C) Flow cytometric evaluation of HL cells stained with 0.25 or 1 µm DiO compared to unstained cells (Ctrl). (D) EV-uptake in fibroblasts cells studied via IF: cytoplasm was stained with cell mask deep red (red), nuclei with Hoechst and HL-derived EVs with DiO (green). Depicted are three representative pictures from upper, middle, and bottom section of a fibroblast. [file image_1.tif]

# Supplementary figure 2

A

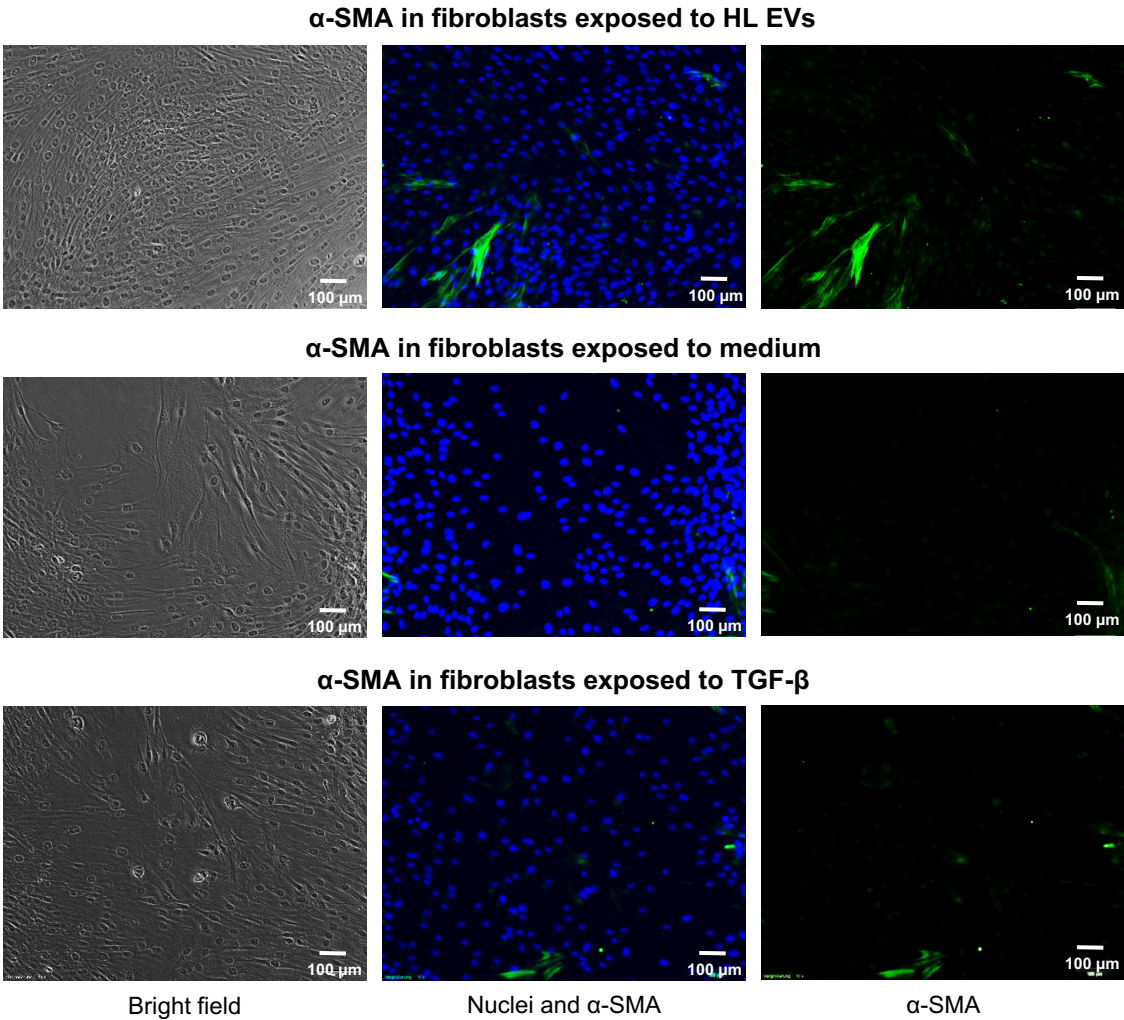

B

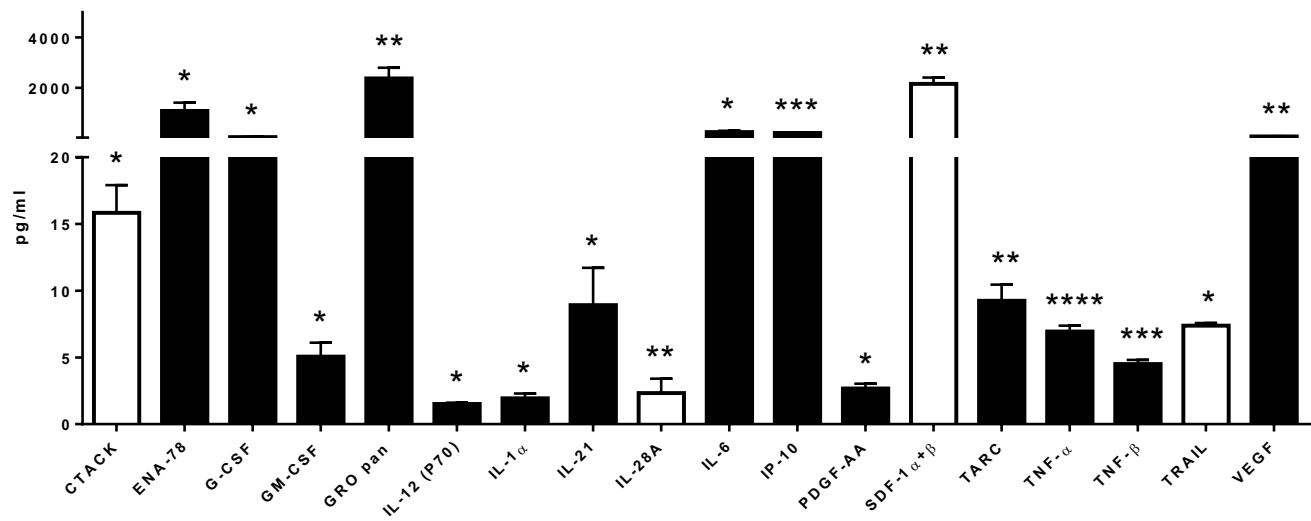

Supplement: Figure S2 — (A) Representative bright field and fluorescence pictures of a scratch assay after 24 h exposure of fibroblasts to 100 µg/ml Hodgkin lymphoma (HL) extracellular vesicles (EVs), medium or 5 ng/ml TGF-β as a positive control. Nuclei are depicted in blue and abundance of α-SMA in green. Microscopic assessment of α-SMA was performed for all three independent scratch assays summarized in Figure 2B. (B) Statistical evaluation of the Human 64-Plex Chemokine Array. Abundance of chemokines/cytokines after 24 h in the supernatant of fibroblasts under influence of HL EVs compared to cells incubated with normal medium (white bars: decrease, black bars: increase). Statistical differences were determined with a Student’s t-test (mean + SEM of three independent replicates; *p ≥ 0.05; **p ≥ 0.01; ***p ≥ 0.001; ****p ≥ 0.0001). [file image_2.PDF]

# Supplementary figure 3

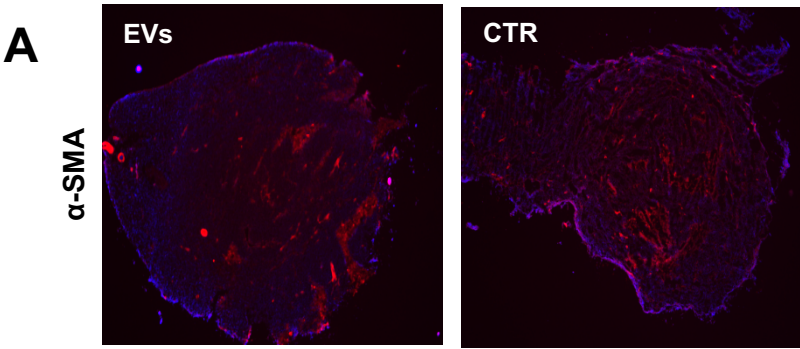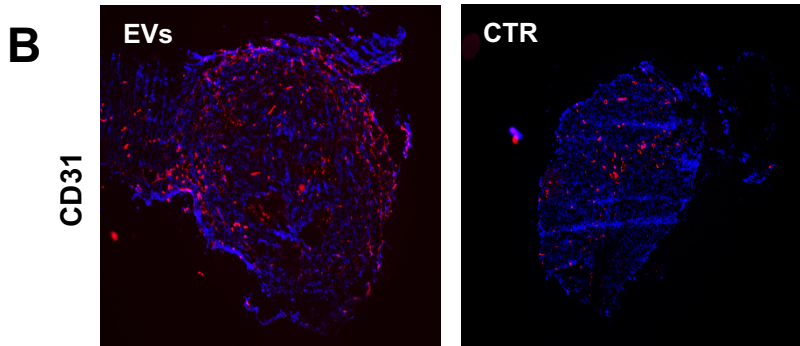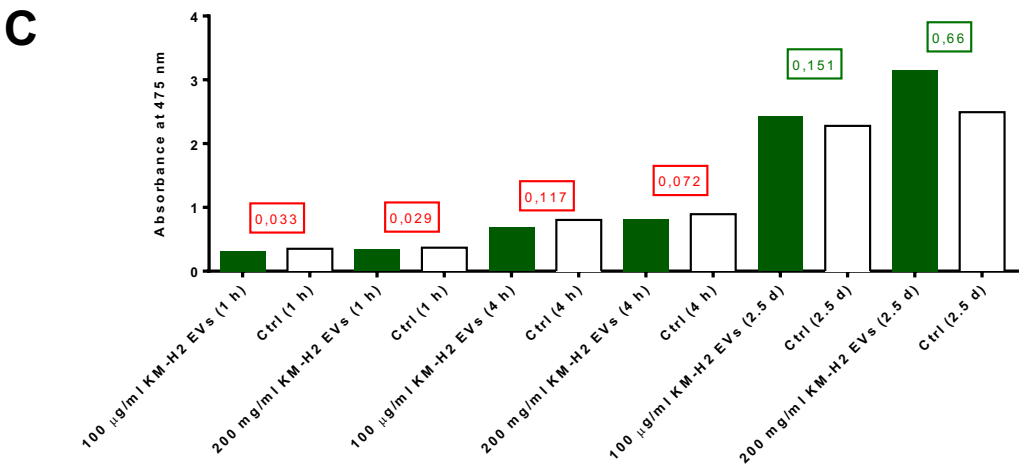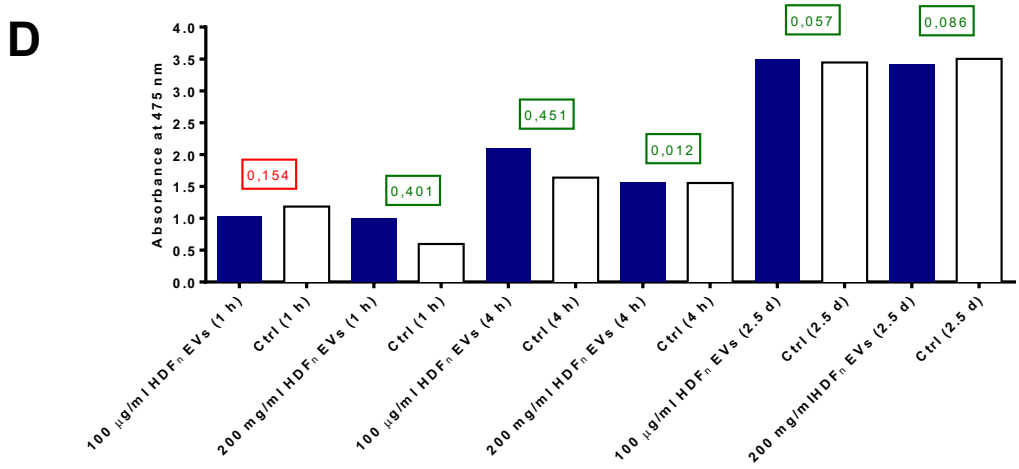

Supplement: Figure S3 — Representative pictures of tumor tissue sections of EV-treated animals and control animals stained for (A) α-SMA or (B) CD31 (red), nuclei were stained with DAPI (blue). Staining was performed for four animals per group. (C) Cell proliferation (XTT assay) of HDFn cells treated with 100 or 200 µg/ml KM-H2 extracellular vesicles (EVs) for the indicated time periods and (D) KM-H2 cells exposed to 100 or 200 µg/ml HDFn EVs over the indicated time course. Depicted is one experiment per cell line. The red numbers display a decrease of cell proliferation compared to the PBS-treated control, whereas green numbers express an increase of proliferation after exposure to EVs. Absorbance was measured at 475 nm with 660 nm as reference wave length. [file image_3.PDF]

## Supplementary figure 4

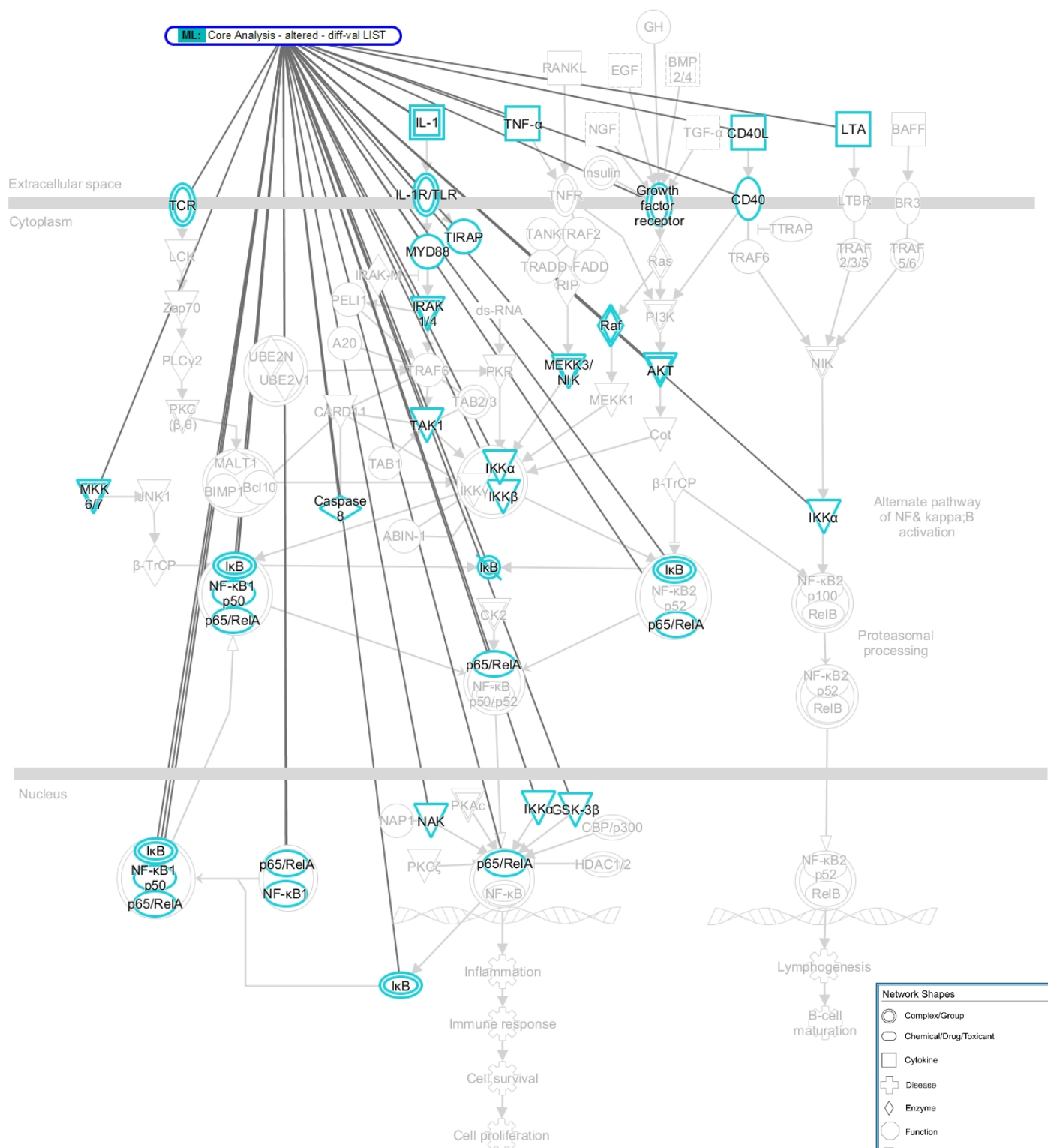

Supplement: Figure S4 — Pathway analysis of proteomics data obtained from analysis of Hodgkin lymphoma extracellular vesicles using the ingenuity pathway analysis (IPA) tool from QIAGEN (IPA Summer Release 2015, QIAGEN Bioinformatics). [file image_4.PDF]
